# Oxygen-enhanced brain MRI using dynamic gradient-echo and MR fingerprinting

**DOI:** 10.1186/s41747-026-00836-6

**Published:** 2026-11-06

**Authors:** Yasutaka Fushimi, Sachi Okuchi, Yusuke Utsunomiya, Satoshi Nakajima, Akihiko Sakata, Takayuki Yamamoto, Sonoko Oshima, Congyu Liao, Hirohiko Imai, Yuji Nakamoto

**Affiliations:** 1 Biomedical Imaging Research Center, University of Fukui, 23-3 Shimoaizuki, Matsuoka, Eiheiji-cho, Yoshida-gun, Fukui, 910-1193 Japan; 2 Department of Diagnostic Imaging and Nuclear Medicine, Graduate School of Medicine, Kyoto University, 54 Shogoin Kawaharacho, Sakyoku, Kyoto, 6068507 Japan; 3 Neuroimaging Technology Research Center, Department of Radiology and Biomedical Imaging, University of California, San Francisco, CA USA; 4 Department of Informatics, Graduate School of Informatics, Kyoto University, Yoshida-Honmachi, Sakyo-ku, Kyoto, 606-8501 Japan; 5 Innovation Research Center for Quantum Medicine, Gifu University School of Medicine, 1-1 Yanagido, Gifu, 501-1194 Japan

**Keywords:** Cerebrospinal fluid, Gray matter, Magnetic resonance imaging, Oxygen, White matter

## Abstract

**Objectives:**

Oxygen-enhanced brain magnetic resonance imaging (MRI) using dynamic three-dimensional (3D) gradient-echo and MR fingerprinting (MRF) was applied to evaluate oxygen transit from blood to cerebrospinal fluid (CSF).

**Materials and methods:**

Twenty healthy participants, enrolled between December 2023 and March 2024, underwent 3-T MRI including Pre-oxygen inhalation 3D MRF (pre-O₂ MRF), dynamic 3D gradient-echo (volumetric interpolated breath-hold examination, VIBE) during oxygen inhalation, and post–oxygen inhalation 3D MRF (post-O₂ MRF). Pre-O₂ and post-O₂ MRF each consisted of two acquisitions. Signal intensity on dynamic VIBE and T1 relaxation times from MRF were evaluated in gray matter (GM), white matter (WM), surface CSF, and ventricles using volumes of interest (VOIs).

**Results:**

Dynamic 3D VIBE demonstrated a rapid signal increase in surface CSF immediately after oxygen inhalation, with a smaller increase in GM. Post-oxygen inhalation VIBE signal was significantly higher than pre-oxygen inhalation signal in surface CSF (*p* < 0.001) and GM (*p* < 0.001). T1 relaxation times differed significantly across phases for GM, WM, and surface CSF (*p* < 0.001) as well as for ventricles (*p* = 0.024). Large effect sizes were observed for surface CSF (Cohen *dz* = 4.15) and GM (Cohen *dz* = 1.31) in dynamic 3D VIBE, as well as for surface CSF (Cohen *f* = 1.31) and GM (Cohen *f* = 0.73) in T1 relaxation time measurement, suggesting substantial physiological changes following oxygen inhalation.

**Conclusion:**

Dynamic 3D VIBE images visualized oxygen inhalation-related signal change in the surface CSF, which were quantitatively validated by T1 relaxation time reductions derived from 3D MRF.

**Key Points:**

***Question*** Can noninvasive oxygen-enhanced brain MRI visualize and quantify oxygen-related signal changes in cerebrospinal fluid using dynamic 3D gradient-echo and 3D MR fingerprinting?

***Findings*** Dynamic 3D gradient-echo showed rapid surface CSF signal increases after oxygen inhalation, accompanied by significant T1 reductions on 3D MR fingerprinting.

***Relevance statement*** This noninvasive oxygen-enhanced MR approach may provide a clinically feasible method for assessing oxygen transit into CSF and could support future investigations of human CSF dynamics. Dynamic 3D gradient-echo images visualized oxygen inhalation-related signal change in the surface CSF, which were quantitatively validated by T1 relaxation time reductions derived from 3D MRF.

**Graphical Abstract:**

**Figure:**
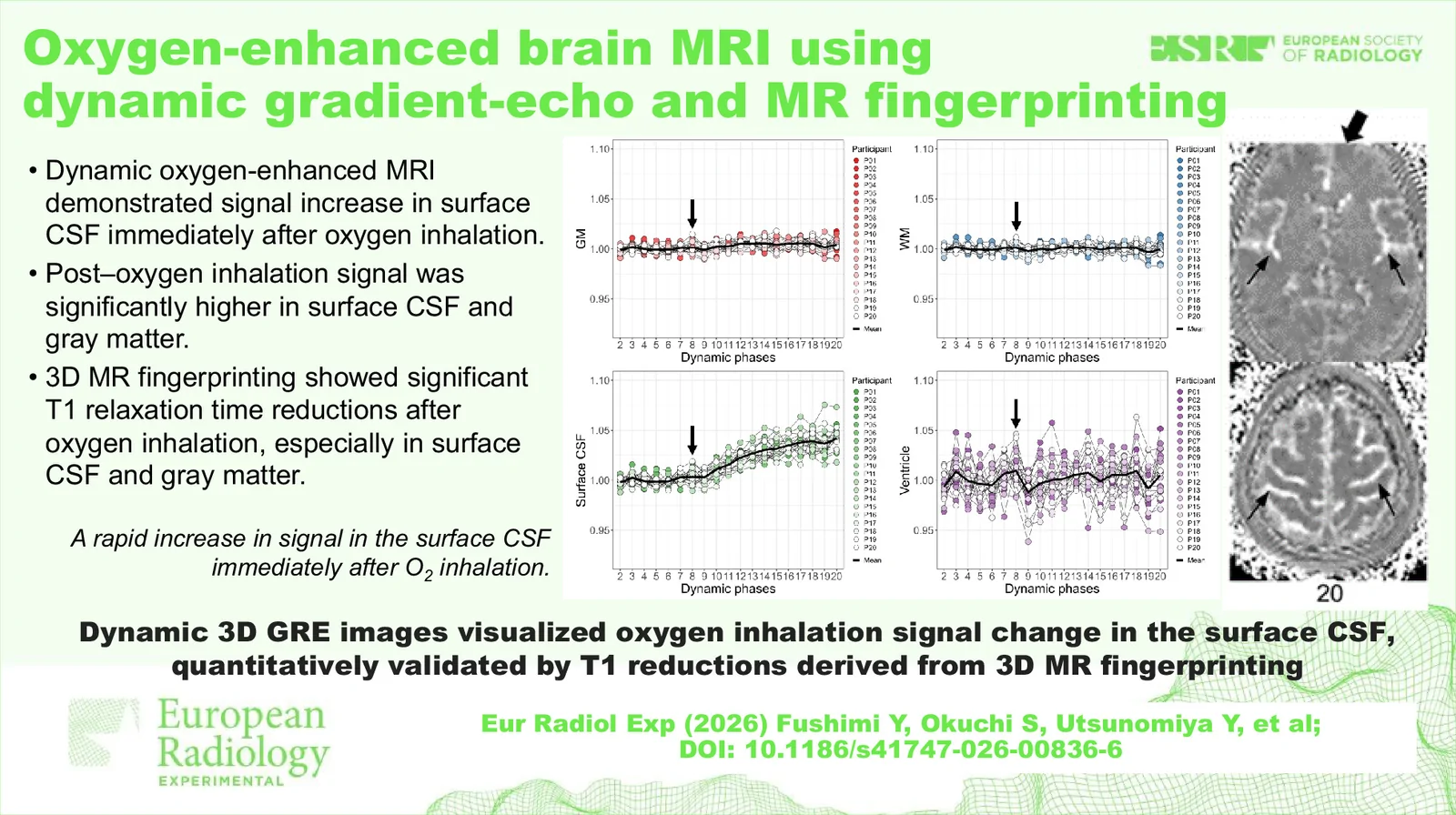

## Introduction

Recently, cerebrospinal fluid (CSF) dynamics have attracted growing attention in the context of the glymphatic system, which allows CSF to permeate the brain parenchyma while bypassing the blood-brain barrier, thereby facilitating the exchange and clearance of interstitial fluid (ISF) and solutes toward the perivascular spaces surrounding veins [1]. Various magnetic resonance imaging (MRI) studies focusing on CSF dynamics have been conducted. Intrathecal injection of gadolinium-based contrast agents for healthy subjects and patients with idiopathic normal pressure hydrocephalus showed that centripetal pattern enhancement within the brain parenchyma was observed a few hours after tracer injection [2, 3]. Gadolinium tracer had been cleared from the brain within 4 weeks after intrathecal administration [2]. However, intrathecal injection of gadolinium-based contrast agents is not permitted globally due to associated health risks [4, 5]. Alternative approaches have also been explored, such as indirect kinetic analysis of 17O-labeled water tracer, which is feasible in the differentiation of idiopathic normal pressure hydrocephalus and possible Alzheimer's disease [6].

Oxygen-enhanced MRI (OE-MRI) also showed signal changes in CSF due to the T1 shortening effect of dissolved oxygen during oxygen inhalation. Inhalation of increased levels of oxygen may lead to qualitatively detectable CSF hyperintensity on fluid-attenuated inversion-recovery images of healthy volunteers [7]. Single-slice dynamic inversion-recovery imaging also demonstrated CSF signal change, demonstrating that the signal change in the surface CSF was larger than the ventricles [8]. Oxygen content has been estimated using three-dimensional (3D) inversion-recovery fast spin echo sequences [9]. Gradient-echo imaging with a slice thickness of 6 mm [10] revealed that the T1-weighted oxygenation response was distributed throughout all tissues in the brain, with the exception of the ventricles. Previous studies showed that a qualitative signal change in CSF after oxygen inhalation was immediately demonstrated using gradient-echo sequences with a focused slab [10].

With the development of quantitative MRI, T1 relaxation time can be obtained with high spatial resolution in clinically feasible scan time using MR fingerprinting (MRF) [11–15]. Although MRF is optimized for brain parenchyma, and conventional MRF dictionaries are optimized for brain parenchyma, the dictionary parameter range can be expanded to encompass the relaxation properties of water and CSF [16]. We hypothesized that quantitative MRI could provide complementary information to qualitative MRI in OE-MRI. Furthermore, we hypothesized that dynamic gradient-echo imaging could elucidate the temporal characteristics of oxygen transit from blood to CSF. To test these hypotheses, we aimed to establish a noninvasive OE brain MRI protocol using 3D MRF in combination with 3D dynamic gradient-echo imaging.

## Materials and methods

### Participants

This prospective study was approved by our institutional review board. Between December 2023 and March 2024, 20 healthy participants were enrolled, and written informed consent was obtained from all participants.

### MRI protocol

All participants underwent 3-T MRI (Magnetom Prisma; Siemens Healthineers) using a 20-channel receive-only head and neck coil.

**Fig. 1:**
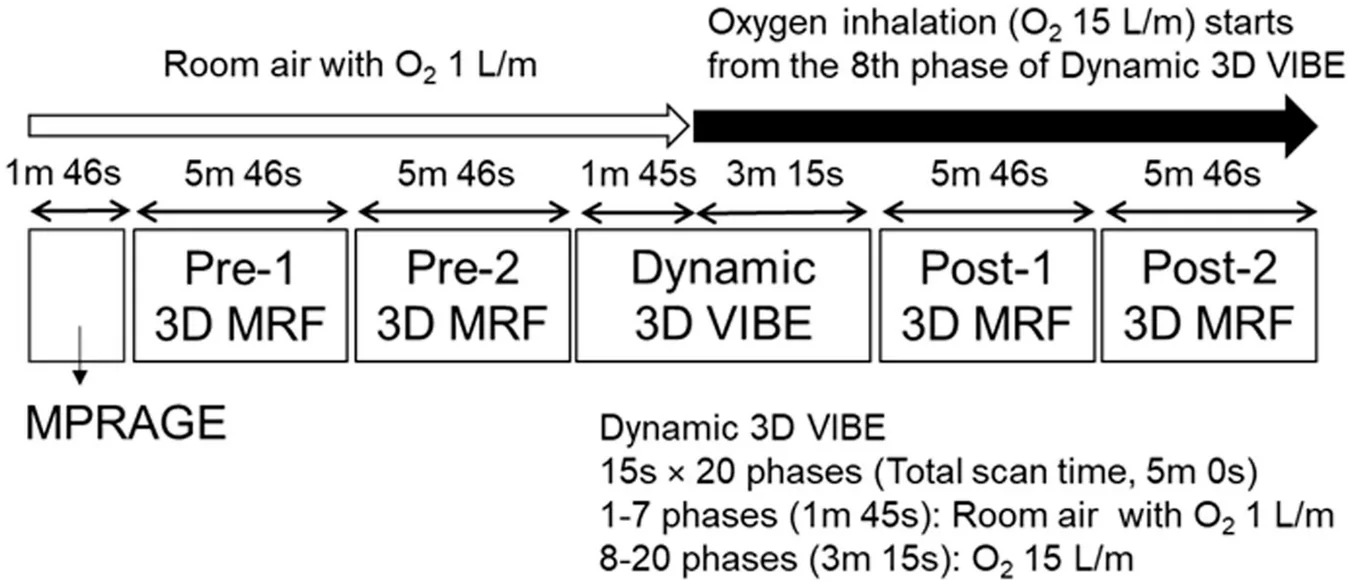
Study protocol and sequence timeline. All participants (*n* = 20) underwent Pre-O_2_ 3D MRF (Pre-1, Pre-2), Dynamic 3D VIBE, and Post-O_2_ 3D MRF (Post-1, Post-2). Oxygen inhalation (15 L/min) starts from the 8th measurement of Dynamic 3D VIBE. Prior to oxygen administration, participants breathed room air with minimal oxygen flow (1 L/min) to reduce rebreathing within the mask. The acquisition time of 1 measurement VIBE is 15 s. *MRF*, Magnetic resonance fingerprinting; *VIBE*, Volumetric Interpolated Breath-hold Examination

All participants underwent Pre-oxygen-inhalation 3D MRF (Pre-O₂ MRF), dynamic oxygen-inhalation 3D gradient-echo (volumetric interpolated breath-hold examination, VIBE), and Post-O₂ MRF. Pre-O₂ MRF and Post-O₂ MRF consisted of two measurements of 3D MRF, namely Pre-1, Pre-2, Post-1, and Post-2 (Fig. 1). Note that pre-O_2_ represents a low-flow supplemental-O_2_ baseline rather than room-air breathing. 3D magnetization-prepared rapid acquisition with gradient echo (MPRAGE) was also obtained for the post-processing.

#### Dynamic oxygen-inhalation 3D VIBE

The technical parameters of the 3D VIBE sequence were as follows: repetition time 4.25 ms; echo time 1.67 ms; flip angle 15°; field of view 256 × 256 mm^2^; resolution 128 × 128; voxel size: 2 × 2 × 2 mm^3^; generalized autocalibrating partially parallel acquisitions–GRAPPA 2 ×  2; acquisition time 15 s; 20 measurements, total scan time 5:0 min:s). At the start of the 8th measurement, oxygen inhalation at a flow rate of 15 L/min was initiated using a reservoir mask (Intersurgical, Berkshire, United Kingdom) and was maintained during the subsequent 3D MRF acquisitions (Post-1 and Post-2). Before the initiation of 15 L/min oxygen inhalation, participants breathed room air through air vents while receiving 1 L/min of oxygen (Fig. 1).

#### 3D MRF

Pre-1, Pre-2, Post-1, and Post-2 3D MRF were obtained in this study. Multiaxis spiral projection imaging was used for 3D MRF to achieve fast quantitative imaging for a whole brain scan [12–14].

An isotropic spatial resolution of 1 × 1 × 1 mm^3^ was achieved for the identified parametric maps in a field of view of 240 × 240 × 240 mm^3^. The acquisition time was 5:46 min:s. The MRF dictionary was adjusted to cover T1 relaxation times ranging from 0 to 5,000 ms in order to include water and CSF. A total of 500 repetition times were acquired for each acquisition group. The entire acquisition duration consists of a total of 30 acquisition groups. At the beginning of each acquisition group, the sequence was prepared by an adiabatic inversion pulse with an inversion time TI of 20 ms. Flip angles varied from 5° to 82° in a sinusoidal pattern. The repetition time and echo time were kept to 16 ms and 2.5 ms, respectively, determined by the duration of the radiofrequency pulse, encoding gradient and dephasing gradient. A variable-density spiral *k*-space sampling trajectory was used for data acquisition, with zero-moment nulling. Image reconstruction was performed on a Linux (Ubuntu 19.04) CPU: Intel Core i9-10920X @ 3.50 GHz Memory: 128 GB random access memory using MATLAB R2019b (MathWorks). Data acquired from the same time point index of all acquisition groups were combined directly. Next, the data were summed with a sliding-window implementation [13] to fill a sphere-like *k*-space, producing image frames, which achieved a significantly improved signal-to-noise ratio and reduced undersampling artifacts. Then, the 3D inverse nonuniform fast Fourier transform, 3D-INUFFT, operator [17, 18] was applied to the sliding-windowed data to reconstruct mixed-contrast and approximately full-sampled images. The sensitivity profiles were estimated using the Eigenvalue-based iterative Self-consistent Parallel Imaging Reconstruction–ESPIRiT method [19] for individual coils, and then the data from each coil were multiplied by the corresponding complex conjugate of the channel sensitivity profile. Finally, template match of 3D image frames was performed using precalculated MRF dictionary voxel-wise.

#### 3D MPRAGE

This sequence was also obtained for the post-processing, with the following technical parameters: repetition time 2,300 ms; echo time 3.31 ms; inversion time 922 ms; flip angle 9°; bandwidth 130 Hz/pixel; isotropic voxel of 0.9 × 0.9 × 0.9 mm^3^; 240 slices. For acceleration, 24 reference lines were acquired in the phase-encoding direction, and WaveCaipi 6× was used. The acquisition time was 1:46 min:s.

### Postprocessing

3D VIBE images were registered to the mean of each subject by using the realign function of SPM12 (https://www.fil.ion.ucl.ac.uk/spm/software/spm12/) with Matlab 2019b (The MathWorks, Inc.). MPRAGE images were registered to 3D VIBE and 3D-MRF images. Brain segmentation was performed for MPRAGE, and binary masks of gray matter (GM), white matter (WM), surface of CSF space, and ventricles were created by using a threshold of 0.3. These masks were used as 3D volumes of interest VOIs (Supplementary Fig. S1).

The first 3D VIBE acquisition (VIBE_1st_) was excluded from the analysis to avoid signal instability, as is commonly done in functional MRI analysis. A single measurement (at 15 s) of VIBE corresponds to multiple functional MRI repetition times (*e.g.*, 2,000 ms). VIBE_Nst_ images were divided by VIBE_2nd_ images (VIBE_Nst_/VIBE_2nd_). The VIBE_2nd_ images were used as the reference to minimize initial signal instability. A dynamic intensity curve was drawn for visualization of OE-MRI, so that pre-O_2_ (average of VIBE_3rd_/VIBE_2nd_ to VIBE_7th_/VIBE_2nd_) was set to 1 for each VOI of each participant to facilitate better visualization.

Pre-O_2_ VIBE (average of VIBE_2nd_ to VIBE_7th_) and Post-O_2_ VIBE (average of VIBE_9th_ to VIBE_20th_) were compared by the VOIs. Slope of linear regression model for pre-O_2_ VIBE and Post-O_2_ VIBE was also performed to evaluate the drift for pre-O_2_ and the signal increase after oxygen inhalation for Post-O_2_. Coefficients of variation of pre-O_2_ (VIBE_3rd_/VIBE_2nd_ to VIBE_7th_/VIBE_2nd_), and subject-level Δ(Post *minus* Pre) were evaluated.

All aforementioned calculations were performed in the native space. To create average images of VIBE_Nst_/VIBE_2nd_ images of all subjects for better visualization, 3D VIBE images were spatially normalized to Montreal Neurological Institute–MNI space by using MPRAGE with Diffeomorphic Anatomical Registration Through Exponentiated Lie Algebra–DARTEL [20].

Offline reconstruction was performed to create T1 maps for 3D MRF. The dictionary size of MRF was adjusted to cover water as 0–5,000 ms for the T1 relaxation time. T1 relaxation time of each phase (Pre-1, Pre-2, Post-1, and Post-2) were evaluated by each 3D VOI [12–14].

Supplementary analyses using one-voxel eroded VOIs were performed. To minimize the partial volume effect of these 3D VOIs, one-voxel eroded VOIs were created from the original VOIs for supplementary analyses (Supplementary Fig. S2). The same post-processing for 3D VIBE and 3D-MRF was performed using these one-voxel-eroded VOIs.

Supplementary analyses of 3D-MRF were performed. R1 relaxation time can be obtained from 3D-MRF in addition to the T1 relaxation time. R1 relaxation time of each phase (Pre-1, Pre-2, Post-1, and Post-2) was evaluated for each 3D VOI. Ratios of T1 relaxation times relative to Pre-1 were also calculated for each dynamic phase (Pre-1/Pre-1, Pre-2/Pre-1, Post-1/Pre-1, and Post-2/Pre1).

In addition, Cramer-Rao lower bound (CRLB)-based sensitivity analysis [21] using the current sequence was performed to evaluate precision over a range of T1/T2 values.

### Statistical analysis

MedCalc version 23.4.5 (MedCalc Software Ltd.), R Studio and R [22, 23] were used for statistical analysis. Paired two groups (pre-O_2_ VIBE and Post-O_2_ VIBE) were compared by using the Wilcoxon signed-rank test if normal distribution was not found (Shapiro–Wilk test). Cohen’s *dz* was calculated to quantify the within-subject effect size between the Pre- and Post-O_2_VIBE for each VOI. Correction for multiple comparisons across VOIs was performed using the false discovery rate (FDR) method.

Four groups (Pre-1, Pre-2, Post-1, and Post-2) were compared by using the Friedman test if the normal distribution was not found. When a significant result was found *(p* < 0.05), post hoc multiple comparisons were conducted using the procedure described by Conover [24]. Cohen’s *f* was calculated to quantify the within-subject effect size across the four conditions (Pre-1, Pre-2, Post-1, and Post-2) for each VOI in 3D-MRF, based on repeated-measures analysis of variance–ANOVA. Intraclass correlation coefficients (ICC) and a paired *t*-test were calculated to evaluate the test–retest reliability of T1 relaxation times between Pre-1 and Pre-2.

R Studio and R were used for the visualization of graphs [22, 23].

## Results

### Study population

A total of 20 participants underwent OE-MRI (mean age ± standard deviation, 74.4 ± 4.8 years, 11 females, 9 males). All participants completed the MRI without any complications.

### 3D VIBE

**Fig. 2:**
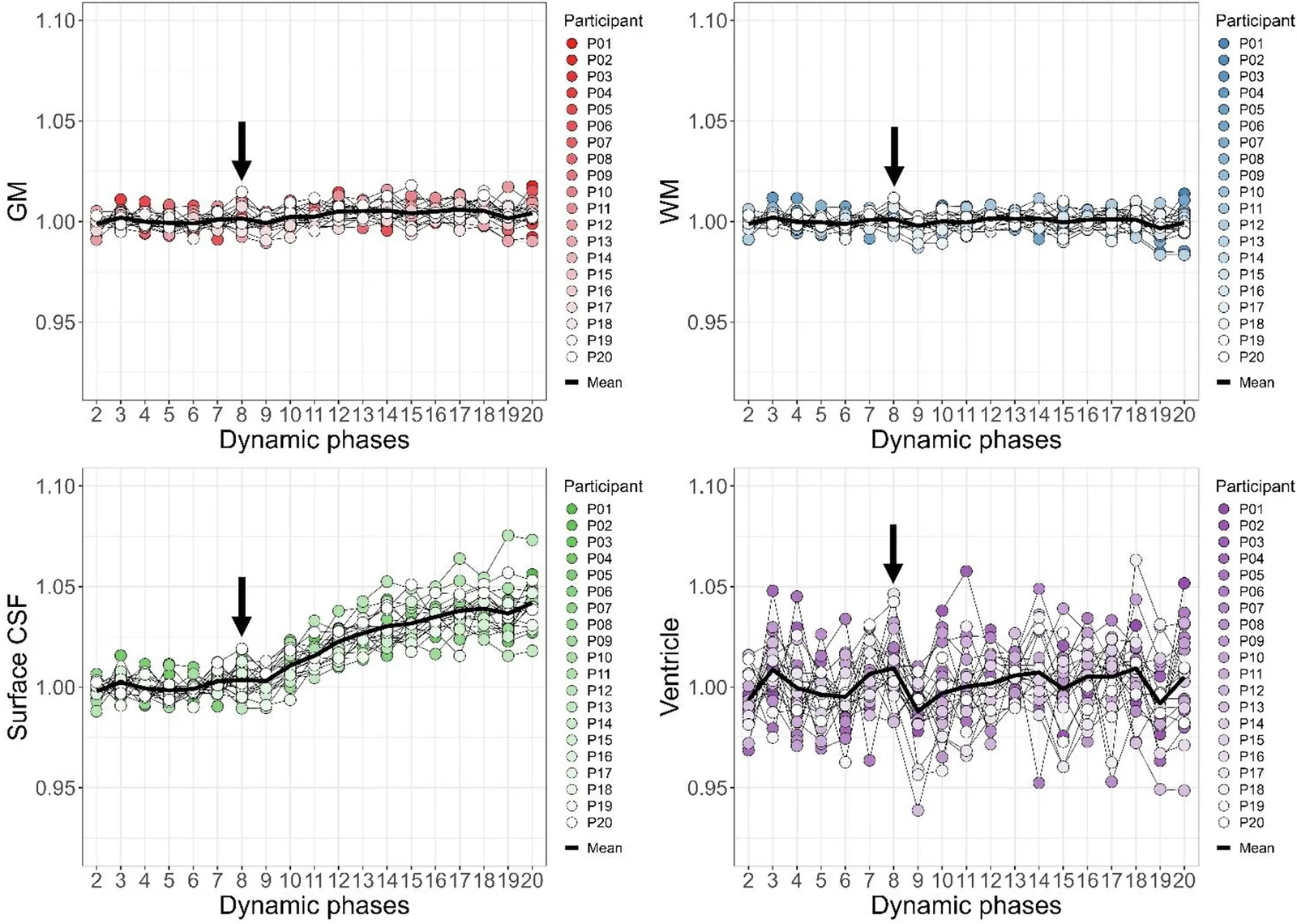
Dynamic changes in signal intensity on 3D VIBE. Temporal changes in the signal intensity ratio of VIBE_Nst_/VIBE_2nd_ are shown across VOIs. Oxygen inhalation was initiated at the 8th measurement (arrow). A rapid increase in signal was observed in the surface CSF immediately after O_2_ inhalation. A slight increase in signal was also observed in GM. Each circle represents the corresponding Participant 1–20. Signals of VIBE_Nst_/VIBE_2nd_ were normalized so that Pre-O_2_ (average of VIBE_3rd_/VIBE_2nd_ to VIBE_7th_/VIBE_2nd_) was set to 1 for each VOI of each participant to facilitate better visualization. VIBE, Volumetric Interpolated Breath-hold Examination; VOI, Volume of interest

**Fig. 3:**
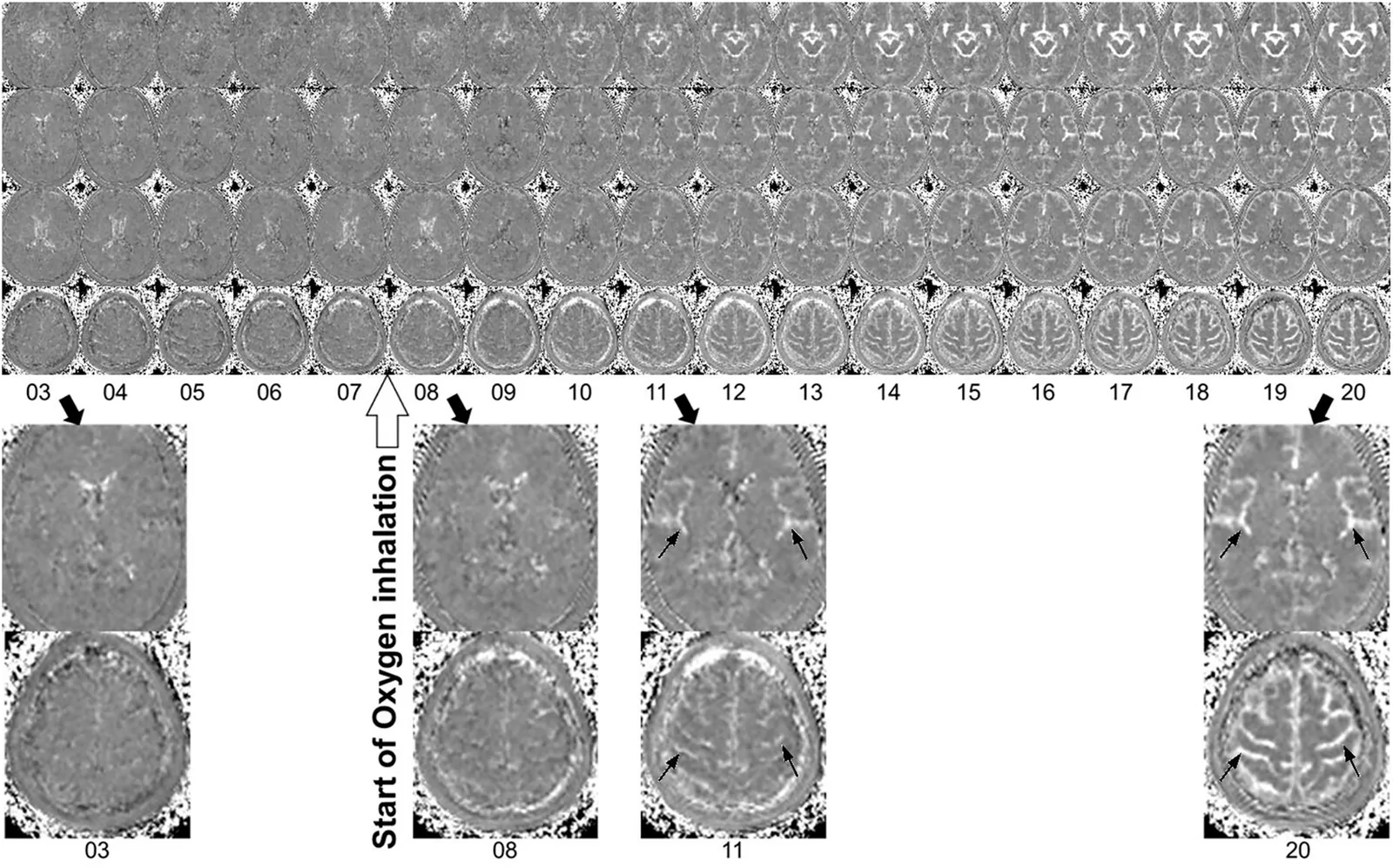
Representative mean images of normalized signal intensity (VIBE_Nst_/VIBE_2nd_) averaged across all participants are shown in Montreal Neurological Institute–MNI space. Signal increase of CSF space (black arrows) is visible in the surface CSF just after O_2_ inhalation. Signals of VIBE_Nst_/VIBE_2nd_ were normalized so that Pre-O_2_ (average of VIBE_3rd_/VIBE_2nd_ to VIBE_7th_/VIBE_2nd_) was set to 1 for each VOI of each participant to facilitate better visualization. VIBE, Volumetric Interpolated Breath-hold Examination; VOI, Volume of interest

Dynamic changes of VIBE_Nst_/VIBE_2nd_ were shown (Fig. 2). A rapid signal increase was observed in the surface CSF immediately after oxygen inhalation. No apparent signal increases were observed in WM or ventricles. Dynamic changes of spatially normalized mean VIBE_Nst_/VIBE_2nd_ were also clearly visualized (Fig. 3). Dynamic signal intensity changes on 3D VIBE for GM were presented with a thicker solid line and a narrower y-axis (Supplementary Fig. S3).

**Fig. 4:**
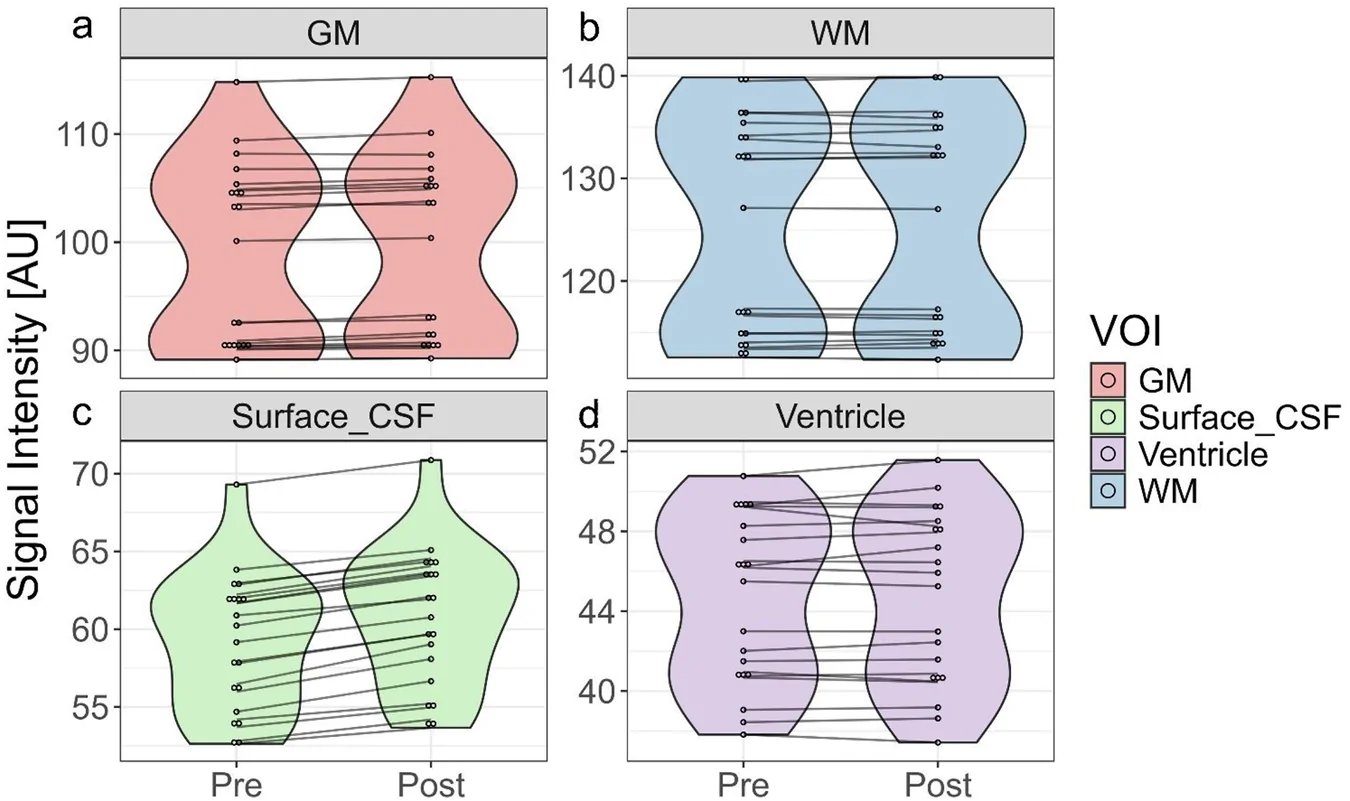
Comparison of signal intensity (arbitrary units) between pre-O_2_ VIBE (average of VIBE_2nd_ to VIBE_7th_) and post-O_2_ VIBE (average of VIBE_9th_ to VIBE_20th_). A slight but significant increase was observed in the surface CSF of post-O_2_ VIBE compared with pre-O_2_ VIBE. A faint but significant increase was also observed in GM, whereas no significant changes were detected in WM or ventricular CSF. CSF, Cerebrospinal fluid; GM, Gray matter; VIBE, Volumetric Interpolated Breath-hold Examination; WM, White matter

Signals in Post-O_2_ VIBE were compared with pre-O_2_ VIBE by each 3D VOI (Table 1 and Fig. 4). Slight but significant signal increase after O_2_ inhalation was observed in surface CSF and GM *(p* < 0.001 for both). A very large effect size was observed in surface CSF (*dz* = 4.15), followed by a large effect in GM (*dz* = 1.31), whereas negligible effects were found in WM *(dz* = 0.01) and ventricles (*dz* = 0.14) (Table 1).

**Table 1:** Signals of Pre-O_2_ and Post-O_2_ VIBE

	GM	WM	Surface CSF	Ventricle
Pre-O_2_ VIBE	98.8 ± 8.2 101.6 [90.6, 105]	125.3 ± 10.3 129.5 [1,14.9, 134.5]	59.0 ± 4.3 59.7 [55.7, 62.1]	44.4 ± 4.1 45.8 [40.9, 48.5]
Post-O_2_ VIBE	99.1 ± 8.2 101.9 [91.2, 105.6]	125.3 ± 10.3 129.5 [115, 134.8]	60.6 ± 4.4 61.3 [57.7, 63.7]	44.5 ± 4.2 45.6 [40.8, 48.3]
*p*-value	< 0.001	0.841	< 0.001	0.701
Cohen *dz*	1.31	0.01	4.15	0.14

Data are presented as mean ± standard deviation and median [interquartile range]. The signal values were expressed in arbitrary units. Note that Pre-O_2_ VIBE represents the average of (VIBE_2nd_ to VIBE_7th_) and post-O_2_ VIBE represents the average of VIBE (average of VIBE_9th_ to VIBE_20th_)

*CSF* Cerebrospinal fluid, *GM* Gray matter, *VIBE* Volumetric Interpolated Breath-hold Examination, *WM* White matter

Coefficients of variation of pre-O_2_ were shown in Supplementary Table S1. They suggest that there existed some drift during the pre-O2 phase; however, the effect was minimal. Slopes of post-O_2_ were shown in Supplementary Fig. S4. Slopes of surface CSF were significantly highest among the four VOIs (Supplementary Table S2). Subject level of signal difference of (post-O2 *minus* pre-O2) of VIBE shows a clear increase of Surface CSF (Supplementary Table S2).

### 3D MRF

**Fig. 5:**
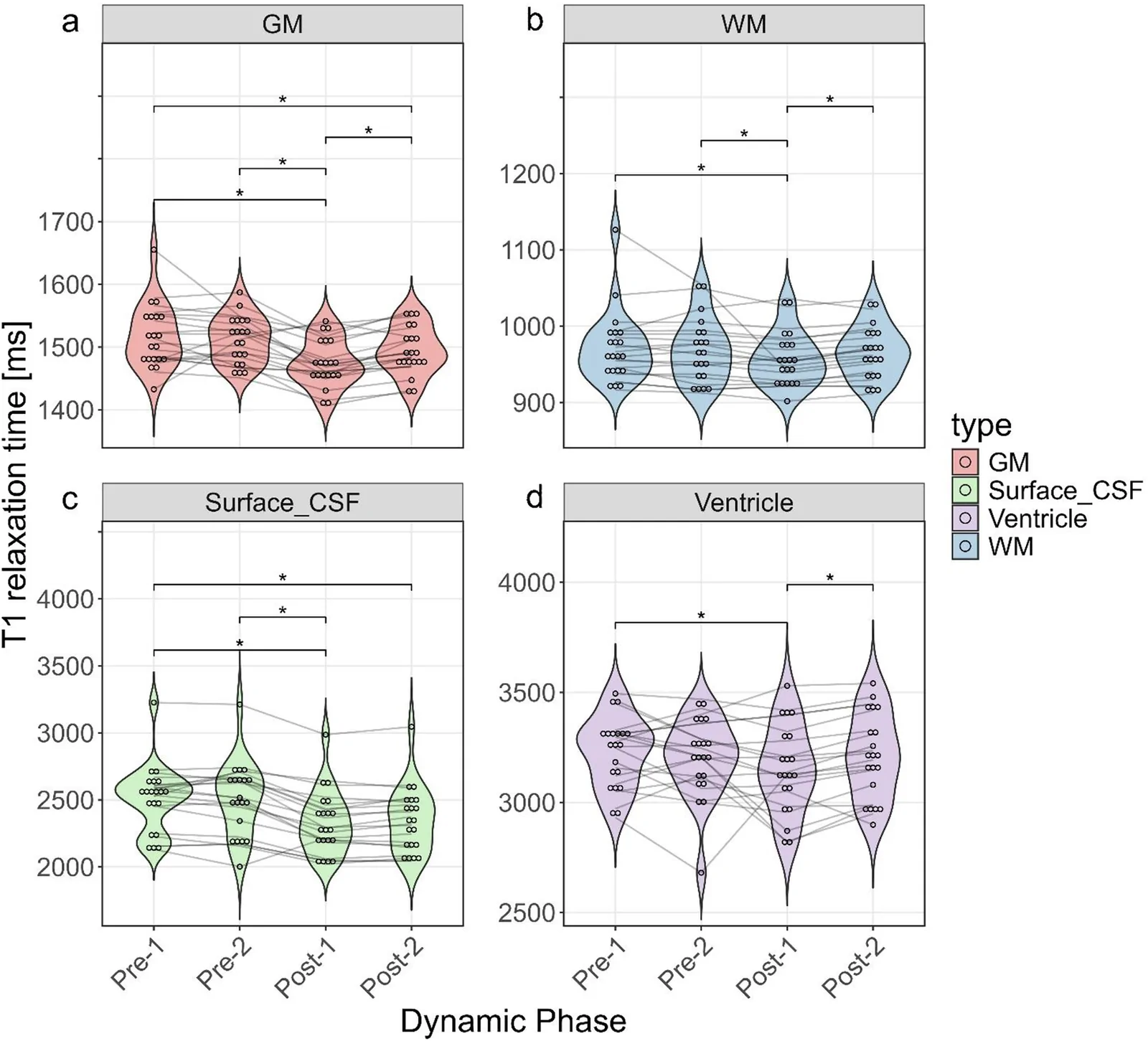
Quantitative T1 relaxation time changes derived from 3D MRF. Box plots show T1 relaxation times [ms] for each VOI across the four dynamic phases (Pre-1, Pre-2, Post-1, and Post-2). Significant T1 reductions were observed after oxygen inhalation, particularly in the surface CSF and GM. Asterisks indicate statistically significant differences between phases based on post hoc multiple comparisons following the Friedman test. CSF, Cerebrospinal fluid; GM, Gray matter; WM, White matter

T1 relaxation times differed significantly across all VOIs during the dynamic phases *(p*-values for GM, WM, surface CSF, and ventricles were < 0.001, <  0.001, < 0.001, and 0.024, respectively; Table 2, Fig. 5). Post hoc analysis using the Conover test revealed the following:

- T1_GM: Post-1 (mean, 1,474.7 ms) was significantly lower than Pre-1 (mean, 1,516.7 ms), Pre-2 (mean, 1,512.0 ms) and Post-2 (mean, 1,495.4 ms) (all *p* < 0.001). Post-2 was significantly lower than Pre-1 *(p* = 0.004), while no significant differences were observed among the remaining comparisons;
- T1_WM: Post-1 (mean, 957.0 ms) had a significantly lower value than Pre-1 (mean, 972.8 ms), Pre-2 (mean, 968.6 ms) (both *p* < 0.001), and Post-2 (mean, 965.5 ms) (*p* = 0.001), while no significant differences were observed among the remaining comparisons;
- T1_Surface CSF: Post-1 (mean, 2,332.7 ms) was significantly lower than Pre-1 (mean, 2,512.4 ms) and Pre-2 (mean, 2,505.9 ms) (all *p* < 0.001). Post-2 (mean, 2,351.3 ms) was significantly lower than Pre-1 (*p* < 0.001), while no significant differences were observed among the remaining comparisons;
- T1_Ventricle: Post-1 (mean, 3,150.6 ms) was significantly lower than Pre-1 (mean, 3,230.4 ms) and Post-2 (mean, 3,211.7 ms) *(p* = 0.012, *p* = 0.004), while no other pairwise differences reached statistical significance.

**Table 2:** T1 relaxation times of each dynamic phase

T1 [ms]	GM	WM	Surface CSF	Ventricle
Pre-1	1,516.7 ± 50.7 1,509.9 [1,482.4, 1,551.3]	972.8 ± 47.4 963.1 [943.7, 990.3]	2,512.4 ± 253.0 2,553.1 [2,402.7, 2,608.6]	3,230.4 ± 158.6 3,264.3 [3,111.4, 3,315.2]
Pre-2	1,512.0 ± 37.3 1,516.0 [1,482.2, 1539.0]	968.6 ± 42.1 965.4 [936.0, 990.8]	2,505.9 ± 276.9 2,515.0 [2,312.6, 2,667.5]	3,200.6 ± 181.1 3,208.4 [3,114.2, 3,305.6]
Post-1	1,474.7 ± 38.0 1,469.3 [1,453.1, 1506.2]	957.0 ± 35.3 953.0 [927.4, 977.6]	2,332.7 ± 239.9 2,293.0 [2,196.3, 2,440.6]	3,150.6 ± 201.8 3,132.0 [3,033.5, 3290.3]
Post-2	1,495.4 ± 38.3 1,490.9 [1,469.8, 1,519.4]	965.5 ± 34.0 965.4 [939.3, 987.1]	2,351.3 ± 246.6 2,347.8 [2,154.4, 2,488.2]	3,211.7 ± 192.9 3,209.5 [3,057.5, 3,349.3]
*p*-value (Friedman)	< 0.001	< 0.001	< 0.001	0.024
Cohen *f*	0.73	0.34	1.31	0.33

Data are presented as mean ± standard deviation and median [interquartile range]

*CSF* Cerebrospinal fluid, *GM* Gray matter, *WM* White matter

### Supplementary analyses using one-voxel eroded VOIs

By using one-voxel eroded VOIs from the original VOIs, dynamic changes of VIBE_Nst_/VIBE_2nd_, comparison of signals between Pre-O_2_ VIBE and Post-O_2_ VIBE (Supplementary Fig. S5). A rapid increase in signal was observed in the surface CSF immediately after O_2_ inhalation (Supplementary Fig. S5). Dynamic signal intensity changes on 3D VIBE for GM were presented with a thicker solid line and a narrower *y*-axis (Supplementary Fig. S6). Signals in Post-O_2_ VIBE were significantly higher than Pre-O_2_ VIBE in 3D VOI of GM and surface CSF (*p*-values for GM and surface CSF were < 0.001 and < 0.001, respectively (Supplementary Table S3).

T1 relaxation times differed significantly across all one-voxel eroded VOIs during the dynamic phases *(p*-values for GM, WM, surface CSF, and ventricles were < 0.001, < 0.001, < 0.001, and 0.032, respectively). The details are described in Supplementary Table S4.

### R1 relaxation times and ratios of T1 relaxation times relative to Pre-1

R1 relaxation times differed significantly across all one-voxel eroded VOIs during the dynamic phases (*p*-values for GM, WM, surface CSF, and ventricles were < 0.001, 0.001, < 0.001, and 0.013, respectively). The details are described in Supplementary Table S5.

Ratios of T1 relaxation time differed significantly across all VOIs during the dynamic phases *(p*-values for GM, WM, surface CSF, and ventricles were < 0.001, < 0.001, < 0.001, and 0.024, respectively). The details are described in Supplementary Table S6.

### Test–retest reliability of 3D MRF

Test–retest reliability was assessed using a two-way random-effects ICC for absolute agreement (ICC(2,1)). The ICC values were 0.825 (95% confidence interval (CI): 0.615–0.926) for gray matter (GM), 0.917 (95% CI: 0.804–0.966) for white matter (WM), 0.963 (95% CI: 0.911–0.985) for surface CSF, and 0.714 (95% CI: 0.415–0.875) for the ventricles. These results indicate good-to-excellent test–retest reliability for GM, WM, and surface CSF, whereas ventricular measurements showed moderate reliability.

### CRLB-based sensitivity analysis

CRLB-based sensitivity analysis using the current sequence was performed (Supplementary Fig. S7). In general, lower CRLB values indicate more precise parameter estimation; however, sensitivity to very long T1/T2 components (*e.g.*, CSF) is inherently limited.

## Discussion

In this study, rapid signal increases during dynamic oxygen inhalation were observed in the surface CSF on 3D VIBE imaging and were statistically significant in both the surface CSF and GM. Large effect sizes were observed for surface CSF (Cohen *dz* = 4.15) and GM (Cohen *dz* = 1.31), indicating a consistent oxygen-related MRI response across participants. It is worth noting that this large effect size primarily reflects the high within-subject consistency of the VIBE signal change across participants, rather than a large absolute signal change, as the mean signal increase in the surface CSF remained modest at approximately 2–3%. In addition, significant reductions in T1 relaxation times derived from 3D MRF were observed after oxygen inhalation across all VOIs, with the most pronounced and sustained changes in the surface CSF and GM, showing large effect sizes of Cohen *f* = 1.31 and 0.73, respectively.

The OE-MRI-related signal changes of 3D VIBE were validated by corresponding T1 relaxation time changes derived from 3D-MRF. Although a significant reduction in T1 relaxation time was confirmed, signal changes of 3D VIBE were subtle compared with previously reported single slice or selective slab sequences [8, 10], partly because 3D VIBE used in this study is a commonly used whole brain gradient-echo based T1 sequence in clinical practices. By creating a ratio image (VIBE_Nst_/VIBE_2nd_), subtle changes become visually apparent. The rapid signal changes observed on dynamic OE-MRI may reflect the rapid transfer of increased intra-arterial dissolved oxygen into the CSF, although the study was not designed to determine whether blood dissolved oxygen enters the brain parenchyma and/or ISF [1, 25]. Therefore, although the observed signal changes may be related to oxygen delivery to CSF-adjacent spaces and GM, these MRI findings should not be interpreted as direct evidence of ISF or CSF clearance [26, 27].

OE-MRI-related changes were observed predominantly in the surface CSF adjacent to the cerebral cortex, followed by GM. The largest reduction in T1 relaxation time was observed in surface CSF, with the second-largest reduction in GM. GM is relatively richer in arterial vasculature than WM [28], and increased influx of intra-arterial dissolved oxygen may contribute to both the T1-weighted signal increase and the reduction in T1 relaxation time observed in GM and surface CSF. Although molecular oxygen is a weak paramagnetic substance, oxygen inhalation reduces deoxyhemoglobin via increased blood oxygenation. Because deoxyhemoglobin is strongly paramagnetic, this leads to reduced magnetic susceptibility differences; however, increased intravoxel dephasing due to increased static field inhomogeneity caused by oxygen-related susceptibility change has also been reported [29]. T2* effects do not directly influence T1 relaxation time; however, both T1 and T2* may be altered by the same underlying physiological factors. In addition, vascular contamination may affect both the T1-weighted signal and the estimated T1 relaxation time, particularly in regions such as surface CSF and GM. Although voxel-based VOI analysis with eroded volumes yielded consistent results, the complete exclusion of leptomeningeal vascular contribution is not possible, and subvoxel vascular contamination cannot be completely eliminated. Inflow effects may also contribute to signal changes, as freshly oxygenated blood entering the imaging slice can increase signal intensity independently of tissue oxygenation. Furthermore, T1 relaxation times and oxygen solubility are temperature-dependent. These complex factors may have affected the present results.

Although changes in T1 relaxation time were also observed in WM and ventricular CSF, these differences were relatively small. WM is also known to be less vascularized and to have slow ISF clearance [2]; therefore, the limited time course of our MRI protocol may have reduced its sensitivity to oxygen-related changes. With regard to the ventricular CSF, the relatively small signal and T1 changes suggest that the present OE-MRI protocol was less sensitive to oxygen-related changes in the ventricular compartment than in the surface CSF. However, this finding should not be interpreted as direct evidence regarding the anatomical source of CSF production or the relative contribution of the choroid plexus. Our results also suggest that simple diffusion of dissolved oxygen from artery to CSF may be limited at least in the artery supplied to the choroid plexus, which is consistent with previous reports of slower mixing in the ventricular compartment in the preclinical study [1] and delayed tracer enrichment observed in the intrathecal gadolinium-based contrast agents administration study [2]. In general, the shift from normoxic to hyperoxic breathing led to vasoconstriction in both the rat (68.5% of the brain) and mouse (77.4%) brain [30]. In humans, phase contrast MR angiography demonstrated that O_2_ administration reduces cerebral blood flow by a mean value of over 20%, with a greater reduction observed in young adults [31]. They demonstrate that, in the absence of systemic inflammation, short-term hyperoxia (100% O_2_ for 2.5 h in mice and 3.5 h in humans) does not result in increased levels of inflammatory cytokines in either mice or healthy volunteers [31]. Furthermore, neutrophil phagocytosis and reactive oxygen species (ROS) generation are unaffected by short-term hyperoxia [32]. However, short-term hyperoxia induced functional and morphological changes in rat hippocampus [33], which suggest that uncontrolled hyperoxia should be avoided even in short-term settings. In addition, CO_2_ concentrations have been shown to increase behind cloth masks, surgical masks, and N95 masks, resulting in mask-induced CO₂ exposure [34]. Since end-tidal CO₂ exposure was not measured in this study, mask-induced CO₂ exposure may have affected the results.

There are several limitations to this study. First, although oxygen inhalation revealed a rapid signal change, a true bolus administration of oxygen was not performed. Oxygen delivery depended on each participant’s respiratory status, although respiration was visually monitored throughout the MRI examination. Administration of oxygenated water would be ideal for bolus evaluation; however, this approach is currently unavailable. Second, the T2 relaxation time was not analyzed. Inhaled oxygen leads to a measurable reduction in T2*, whereas changes in T2 are considered negligible [35]. Third, OE-MRI was performed in the awake state. Because CSF-related physiology may differ between awake and sleep conditions, comparison between awake and sleep conditions may be of interest. Fourth, standard MRF acquisition schemes are primarily optimized for GM and WM, and their sensitivity to very long T1/T2 components (*e.g.*, CSF) is limited. Strictly speaking, only the signal changes of 3D dynamic VIBE in GM were validated by 3D MRF owing to the limited sensitivity of MRF to the longer T1 values in CSF. Fifth, signal changes in GM may be attributable to partial volume effects from adjacent surface CSF. Because GM is contiguous with surface CSF, the measured signal may have been influenced by CSF. Sixth, the relatively small sample and the limited number of elderly participants may restrict the generalizability of the findings. Although they were healthy, they possessed a high level of health literacy and a strong willingness to contribute to medical research. Having previously participated in several MRI studies, they were well-suited to this relatively specialized OE-MRI protocol. To improve generalizability, further studies including participants across a broader age range are warranted. Seventh, neither end-tidal CO₂ monitoring nor arterial oxygen saturation measurement was performed in this study. Lastly, no formal sample size calculation, reproducibility assessment, or control condition using a room air inhalation protocol was included. Although the results obtained from 20 participants were relatively consistent, further studies with larger cohorts are needed to strengthen these findings.

## Conclusion

In conclusion, dynamic 3D VIBE images visualized small but consistent oxygen inhalation-related signal change predominantly in the surface CSF, which were quantitatively supported by T1 relaxation time reductions derived from 3D MRF. These findings suggest that clinically feasible OE-MRI may be sensitive to oxygen-related signal changes in CSF-adjacent spaces. However, the physiological implications of these changes remain uncertain, and further studies incorporating physiological monitoring, control conditions, and dedicated experimental designs are needed before linking these MRI findings to CSF dynamics or clearance pathways.

## Supplementary information

**Additional file 1: Supplementary Table S1.** The coefficients of variation of Pre-O2 and the slopes of the linear regressions for Pre-O2. **Supplementary Table S2.** Subject level of signal difference of (Post-O2 minus Pre-O2) of VIBE **Supplementary Table S3.** Signals of Pre-O2 and Post-O2 VIBE using VOIs eroded by 1 voxel from the original VOIs. **Supplementary Table S4.** T1 relaxation times of each dynamic phase using VOIs eroded by 1 voxel from the original VOIs. **Supplementary Table S5**. R1 relaxation times of each dynamic phase **Supplementary Table S6.** Ratios of T1 relaxation times relative to Pre-1 across dynamic phases. **Supplementary Fig. S1** Representative images of 3D VOIs. CSF Cerebrospinal fluid, GM Gray matter, VOI Volume of interest, WM White matter. **Supplementary Fig. S2** VOIs eroded by 1 voxel from the original VOIs (shown in Supplementary Fig. S1) were additionally created. Representative images of 3D VOIs. CSF Cerebrospinal fluid, GM Gray matter, VOI Volume of interest, WM White matter. **Supplementary Fig. S3** Dynamic signal intensity changes on 3D VIBE for GM were presented with a thicker solid line and a narrower y-axis. A slight signal increase was also observed in GM. GM Gray matter, VIBE Volumetric Interpolated Breath-hold Examination. **Supplementary Fig. S4** Slopes of Post-O2 VIBE (average of VIBE9th to VIBE20th) were shown. Slope of surface CSF was significantly highest among four VOIs. CSF Cerebrospinal fluid, VIBE Volumetric Interpolated Breath-hold Examination, VOI Volume of interest. **Supplementary Fig. S5** Dynamic signal intensity changes on 3D VIBE using VOIs eroded by 1 voxel from the original VOIs. Temporal changes in the signal intensity ratio of VIBENst/VIBE2nd are shown across VOIs. A rapid increase in signal was observed in the surface CSF immediately after O2 inhalation. A slight signal increase was also observed in GM. Each circle represents the corresponding Participant 1-20. CSF Cerebrospinal fluid, GM Gray matter, VIBE Volumetric Interpolated Breath-hold Examination, VOI Volume of interest. **Supplementary Fig. S6.** Dynamic signal intensity changes on 3D VIBE using VOIs eroded by 1 voxel from the original VOIs for GM were presented with a thicker solid line and a narrower y-axis. A slight signal increase was also observed in GM. GM Gray matter, VIBE Volumetric Interpolated Breath-hold Examination, VOI Volume of interest. **Supplementary Fig. S7** Cramér–Rao Lower Bound (CRLB)-based sensitivity analysis using the current sequence was performed to evaluate precision over a range of T1/T2 values, including those typical of CSF. In general, lower CRLB values indicate more precise parameter estimation. To provide a quantitative anchor for the long-T1 regime characteristic of surface CSF, the theoretical precision floor at T1 = 3,000 ms and T2 = 1,000 ms using a conservative target SNR of 10 was shown. Under these conditions, the lower bound for T1 estimation error, CRLB_T1, was 21.26 ms, corresponding to a relative CRLB of 0.71%. The observed Post minus Pre T1 reductions in surface CSF were approximately 150–250 ms, which are about 7–12 times larger than this CRLB-based precision floor. These results indicate that the observed T1 reductions substantially exceed the expected uncertainty from estimation noise and are unlikely to be explained by numerical precision limits alone.

## Data Availability

The datasets used and/or analyzed during the current study are available from the corresponding author on reasonable request.

## Abbreviations

3D: Three-dimensional
CRLB: Cramer-Rao lower bound (sensitivity analysis)
CSF: Cerebrospinal fluid
GM: Gray matter
ISF: Interstitial fluid
MPRAGE: Magnetization-prepared rapid acquisition with gradient echo
MR: Magnetic resonance
MRF: MR fingerprinting
MRI: Magnetic resonance imaging
OE-MRI: Oxygen-enhanced MRI
VIBE: Volumetric interpolated breath-hold examination
VOI: Volume of interest
WM: White matter
