# Supplementary material for: Oxygen-enhanced brain MRI using dynamic gradient-echo and MR fingerprinting
Source: Eur Radiol Exp. 2026 Nov 6;10:174. doi: 10.1186/s41747-026-00836-6 (PMC13743701; doi:10.1186/s41747-026-00836-6)
Supplement: Supplementary file 1 [file 41747_2026_836_MOESM1_ESM.pdf]

# Oxygen-enhanced brain MRI using dynamic gradient-echo and MR fingerprinting
# Electronic Supplementary Material

**Supplementary Table S1** The coefficients of variation of Pre-$O_2$ and the slopes of the linear regressions for Pre-$O_2$

| | GM | WM | Surface | Ventricle |
|---|---|---|---|---|
| CoV | 0.0037 ± 0.0015 | 0.0036 ± 0.0015 | 0.0052 ± 0.0021 | 0.0164 ± 0.0058 |
| Slope ($\times 10^{-3}$) | 0.035 ± 0.743 | 0.008 ± 0.714 | 0.378 ± 0.835 | 0.609 ± 0.294 |

Data are presented as mean ± standard deviation. Note that the coefficient of variation (CoV) of Pre-$O_2$ was calculated as the standard deviation of $VIBE_{3rd}/VIBE_{2nd}$ to $VIBE_{7th}/VIBE_{2nd}$ divided by their mean. The slopes of the linear regressions for Pre-$O_2$ ($VIBE_{3rd}/VIBE_{2nd}$ to $VIBE_{7th}/VIBE_{2nd}$) are shown. The *p*-values for the regressions of each participant were not statistically significant in any VOI. *CoV* Coefficient of variation, *GM* Gray matter, *VIBE* Volumetric Interpolated Breath-hold Examination, *VOI* Volume of interest, *WM* White matter.

**Supplementary Table S2** Subject level of signal difference of (Post-$O_2$ *minus* Pre-$O_2$) of VIBE

| | GM | WM | Surface | Ventricle |
|---|---|---|---|---|
| Post-$O_2$ - Pre-$O_2$ | 0.004 ± 0.003 | 0.000 ± 0.003 | 0.028 ± 0.007 | 0.001 ± 0.010 |

Data are presented as mean ± standard deviation. *GM* Gray matter, *WM* White matter.

**Supplementary Table S3** Signals of Pre-$O_2$ and Post-$O_2$ VIBE using VOIs eroded by 1 voxel from the original VOIs.

| | GM | WM | Surface CSF | Ventricle |
|---|---|---|---|---|
| Pre-$O_2$ VIBE | 100.2 ± 8.7<br>101.4 [92.0, 105.6] | 128.7 ± 10.9<br>132.2 [117.9, 138.3] | 53.5 ± 4.5<br>53.8 [49.1, 56.6] | 35.5 ± 3.4<br>35.2 [32.8, 38.1] |
| Post-$O_2$ VIBE | 100.4 ± 8.7<br>101.6 [92.2, 106.0] | 128.8 ± 10.9<br>132.4 [117.9, 138.7] | 55.2 ± 4.6<br>55.7 [51.1, 58.9] | 35.5 ± 3.6<br>35.7 [32.7, 38.3] |
| *p*-value | <0.001 | 0.409 | <0.001 | 0.898 |
| Cohen *dz* | 0.57 | 0.18 | 3.89 | 0.08 |

Data are presented as mean ± standard deviation and median [interquartile range]. The signal values were expressed in arbitrary unit. Note that Pre-$O_2$ VIBE represents average of ($VIBE_{2nd}$ to $VIBE_{7th}$) and Post-$O_2$ VIBE represents average of VIBE (average of $VIBE_{9th}$ to $VIBE_{20th}$). *CSF* Cerebrospinal fluid, *GM* Gray matter, *VIBE* Volumetric Interpolated Breath-hold Examination, *VOI* Volume of interest, *WM* White matter.

**Supplementary Table S4** T1 relaxation times of each dynamic phase using VOIs eroded by 1 voxel from the original VOIs.

| T1 [ms] | GM | WM | Surface CSF | Ventricle |
|---|---|---|---|---|
| Pre-1 | 1,454.2 ± 56.3<br>1,448.4 [1,433.8, 1,465.9] | 903.7 ± 33.0<br>897.7 [881.2, 920.7] | 3,090.3 ± 317.5<br>3087.9 [2,992.0, 3,252.2] | 3,523.1 ± 169.9<br>3,508.6 [3,392.1, 3,646.3] |
| Pre-2 | 1,447.9 ± 36.8<br>1,445.4 [1,426.7, 1464.3] | 901.9 ± 33.4<br>895.7 [881.1, 918.7] | 3,076.7 ± 349.5<br>3,068.0 [2,897.4, 3,268.7] | 3,488.2 ± 193.9<br>3,514.6 [3,409.1, 3,601.4] |
| Post-1 | 1,419.9 ± 29.2<br>1,419.4 [1,404.1, 1,435.7] | 893.3 ± 31.9<br>889.1 [873.4, 911.2] | 2,845.4 ± 305.6<br>2813.6 [2,663.5, 3,057.5] | 3,418.8 ± 214.6<br>3,425.9 [3,274.3, 3,559.1] |
| Post-2 | 1,435.6 ± 32.7<br>1,443.8 [1,416.1, 1,452.4] | 900.8 ± 29.8<br>898.8 [883.5, 918.9] | 2,858.6 ± 314.5<br>2851.3 [2,629.0, 3041.6] | 3,477.8 ± 220.2<br>3,483.6 [3,308.8, 3,656.1] |
| *p*-value (Friedman) | < 0.001 | < 0.001. | < 0.001 | 0.032 |
| Cohen *f* | 0.66 | 0.38 | 1.54 | 0.37 |

Data are presented as mean ± standard deviation, median [interquartile range]. *CSF* Cerebrospinal fluid, *GM* Gray matter, *WM* White matter.

**3D MRF using eroded VOIs**

T1 relaxation times differed significantly across all VOIs during the dynamic phases (*p*-values for GM, WM, surface CSF, and ventricles were < 0.001, < 0.001, < 0.001, and 0.032, respectively). Post hoc analysis using Conover's test revealed the following:

T1_GM: Post-1 (mean, 1419.9 ms) was significantly lower than Pre-1 (mean, 1454.2 ms) and Pre-2 (mean, 1447.9 ms) (both p < 0.001). Post-2 (mean, 1435.6 ms) was significantly lower than Pre-1 (p = 0.004), while no significant differences were observed among the remaining comparisons.

T1_WM: Post-1 (mean, 893.3 ms) was significantly lower than Pre-1 (mean, 903.7 ms), Pre-2 (mean, 901.9 ms), and Post-2 (mean, 900.8 ms) (all p < 0.001). No significant differences were observed among the remaining comparisons.

T1_Surface CSF: Post-1 (mean, 2845.4 ms) was significantly lower than Pre-1 (mean, 3090.3 ms) and Pre-2 (mean, 3076.7 ms) (both p < 0.001). Post-2 (mean, 2858.6 ms) was significantly lower than Pre-1 and Pre-2 (both p < 0.001), while no significant differences were observed among the remaining comparisons.

T1_Ventricle: Post-1 (mean, 3418.8 ms) was significantly lower than Pre-1 (mean, 3523.1 ms) and Post-2 (mean, 3477.8 ms) (p = 0.009 for both comparisons), while no other pairwise differences reached statistical significance.

**Supplementary Table S5.** R1 relaxation times of each dynamic phase

| R1 [$\times 10^{-3}$/ms] | GM | WM | Surface CSF | Ventricle |
|---|---|---|---|---|
| Pre-1 | 0.724 ± 0.022<br>0.721 [0.709, 0.730] | 1.061 ± 0.037<br>1.065 [1.039, 1.087] | 0.672 ± 0.844<br>0.764 [0.643, 0.938] | 0.362 ± 0.028<br>0.356 [0.347, 0.372] |
| Pre-2 | 0.718 ± 0.050<br>0.721 [0.705, 0.732] | 1.063 ± 0.038<br>1.072 [1.040, 1.089] | 0.718 ± 0.319<br>0.793 [0.681, 0.909] | 0.363 ± 0.028<br>0.358 [0.347, 0.374] |
| Post-1 | 0.748 ± 0.027<br>0.742 [0.729, 0.761] | 1.074 ± 0.037<br>1.083 [1.049, 1.105] | 0.981 ± 0.193<br>0.992 [0.824, 1.146] | 0.366 ± 0.023<br>0.369 [0.347, 0.384] |
| Post-2 | 0.718 ± 0.033<br>0.720 [0.711, 0.730] | 1.064 ± 0.034<br>1.065 [1.040, 1.091] | 0.796 ± 0.232<br>0.720 [0.654, 0.961] | 0.359 ± 0.021<br>0.361 [0.344, 0.373] |
| *p*-value (Friedman) | <0.001 | 0.001 | <0.001 | 0.013 |
| Cohen *f* | 0.73 | 0.34 | 1.31 | 0.33 |

Data are presented as mean ± standard deviation and median [interquartile range]. *CSF* Cerebrospinal fluid, *GM* Gray matter, *WM* White matter.

**3D MRF (R1 relaxation time)**

R1 relaxation rates differed significantly across all VOIs during the dynamic phases (p-values for GM, WM, surface CSF, and ventricles were <0.001, 0.001, <0.001, and 0.013, respectively). Post hoc analysis using Conover's test revealed the following:

R1_GM: Post-1 (mean, 0.748$\times 10^{-3}$ ms) was significantly higher than Pre-1 (mean, 0.724 $\times 10^{-3}$ ms), Pre-2 (mean, 0.718 $\times 10^{-3}$ ms), and Post-2 (mean, 0.718 $\times 10^{-3}$ ms) (all $p < 0.001$). No significant differences were observed among the remaining comparisons.

R1_WM: Post-1 (mean, 1.074$\times 10^{-3}$ ms) was significantly higher than Pre-1 (mean, 1.061$\times 10^{-3}$ ms), Pre-2 (mean, 1.063$\times 10^{-3}$ ms), and Post-2 (mean, 1.064$\times 10^{-3}$ ms) (all $p < 0.001$). No significant differences were observed among the remaining comparisons.

R1_Surface CSF: Post-1 (mean, 0.981$\times 10^{-3}$ ms) was significantly higher than Pre-1 (mean, 0.672$\times 10^{-3}$ ms), Pre-2 (mean, 0.718$\times 10^{-3}$ ms) and Post-2 (mean, 0.796$\times 10^{-3}$ ms) (all $p < 0.001$). No significant differences were observed among the remaining comparisons.

R1_Ventricle: Post-1 (mean, 0.366$\times 10^{-3}$ ms) was significantly higher than Pre-1 (mean, 0.362$\times 10^{-3}$ ms) and Post-2 (mean, 0.359$\times 10^{-3}$ ms) ($p = 0.008$ and $p = 0.003$, respectively). No significant differences were observed among the remaining comparisons.

**Supplementary Table S6** Ratios of T1 relaxation times relative to Pre-1 across dynamic phases

| Ratios | GM | WM | Surface CSF | Ventricle |
|---|---|---|---|---|
| Pre-1 | 1.000 ± 0.000<br>1.000 [1.000, 1.000] | 1.000 ± 0.000<br>1.000 [1.000, 1.000] | 1.000 ± 0.000<br>1.000 [1.000, 1.000] | 1.000 ± 0.000<br>1.000 [1.000, 1.000] |
| Pre-2 | 0.997 ± 0.023<br>0.996 [0.989, 1.006] | 0.996 ± 0.018<br>0.999 [0.991, 1.005] | 0.997 ± 0.032<br>0.998 [0.978, 1.009] | 0.991 ± 0.038<br>0.990 [0.964, 1.020] |
| Post-1 | 0.973 ± 0.035<br>0.973 [0.961, 0.984] | 0.985 ± 0.037<br>0.991 [0.986, 0.993] | 0.930 ± 0.051<br>0.922 [0.911, 0.951] | 0.976 ± 0.054<br>0.973 [0.939, 1.013] |
| Post-2 | 0.987 ± 0.032<br>0.990 [0.975, 0.993] | 0.994 ± 0.036<br>0.999 [0.994, 1.005] | 0.937 ± 0.051<br>0.944 [0.925, 0.953] | 0.995 ± 0.051<br>0.994 [0.961, 1.033] |
| *p*-value (Friedman) | <0.001 | <0.001 | <0.001 | 0.024 |
| Cohen *f* | 0.73 | 0.34 | 1.31 | 0.33 |

Ratios to Pre-1 was calculated: Pre-1/Pre-1, Pre-2/Pre-1, Post-1/Pre-1, and Post-2/Pre1. Data are presented as mean ± standard deviation and median [interquartile range]. *CSF* Cerebrospinal fluid, *GM* Gray matter, *WM* White matter.

**Ratios of T1 relaxation times relative to Pre-1**

Ratios of T1 relaxation time differed significantly across all VOIs during the dynamic phases (p-values for GM, WM, surface CSF, and ventricles were <0.001, <0.001, <0.001, and 0.024, respectively). Post hoc analysis using Conover's test revealed the following:

GM: Post-1 (mean, 0.973) was significantly lower than Pre-1 (mean, 1.000), Pre-2 (mean, 0.997), and Post-2 (mean, 0.987) (all $p < 0.001$). Post-2 was significantly lower than Pre-1 ($p = 0.004$). No significant differences were observed among the remaining comparisons.

WM: Post-1 (mean, 0.985) was significantly lower than Pre-1 (mean, 1.000), Pre-2 (mean, 0.996), and Post-2 (mean, 0.994) ($p < 0.001$, $p < 0.001$, $p = 0.001$). No significant differences were observed among the remaining comparisons.

Surface CSF: Post-1 (mean, 0.930) was significantly lower than Pre-1 (mean, 1.000), Pre-2 (mean, 0.997) (all $p < 0.001$). Post-2 (mean, 0.937) was significantly lower than Pre-1 ($p < 0.001$). No significant differences were observed among the remaining comparisons.

Ventricle: Post-1 (mean, 0.976) was significantly lower than Pre-1 (mean, 1.000) and Post-2 (mean,0.995) ($p = 0.012$ and $p = 0.004$, respectively). No significant differences were observed among the remaining comparisons.

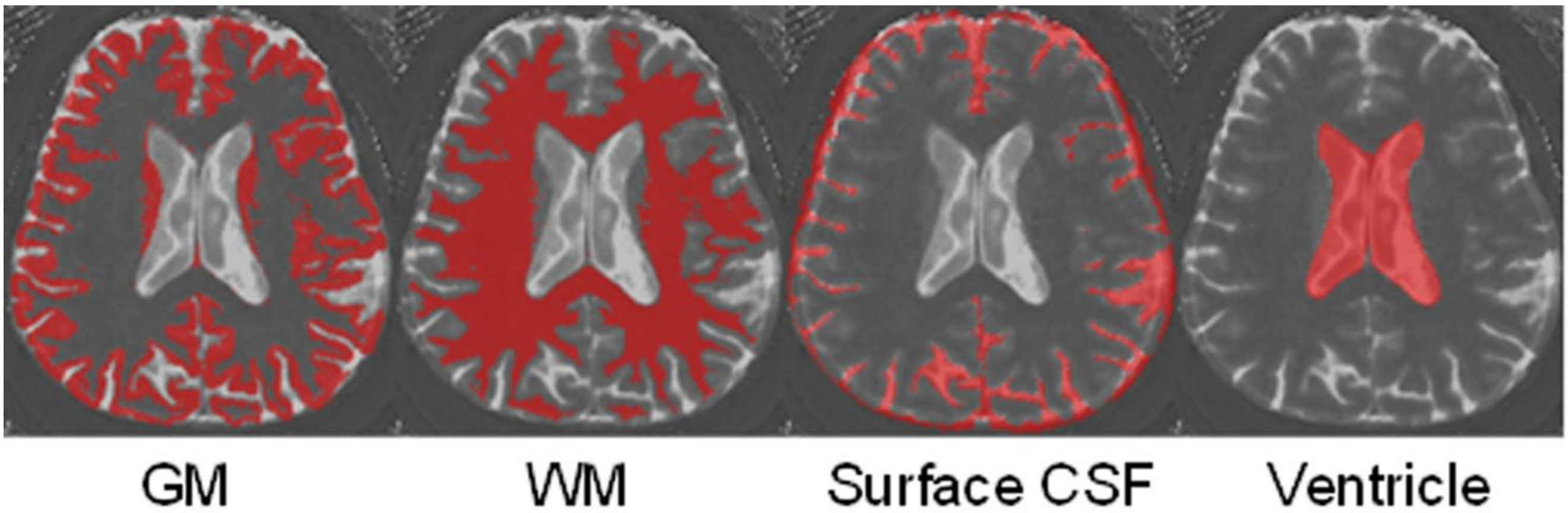


**Supplementary Fig. S1** Representative images of 3D VOIs. *CSF* Cerebrospinal fluid, *GM* Gray matter, *VOI* Volume of interest, *WM* White matter.

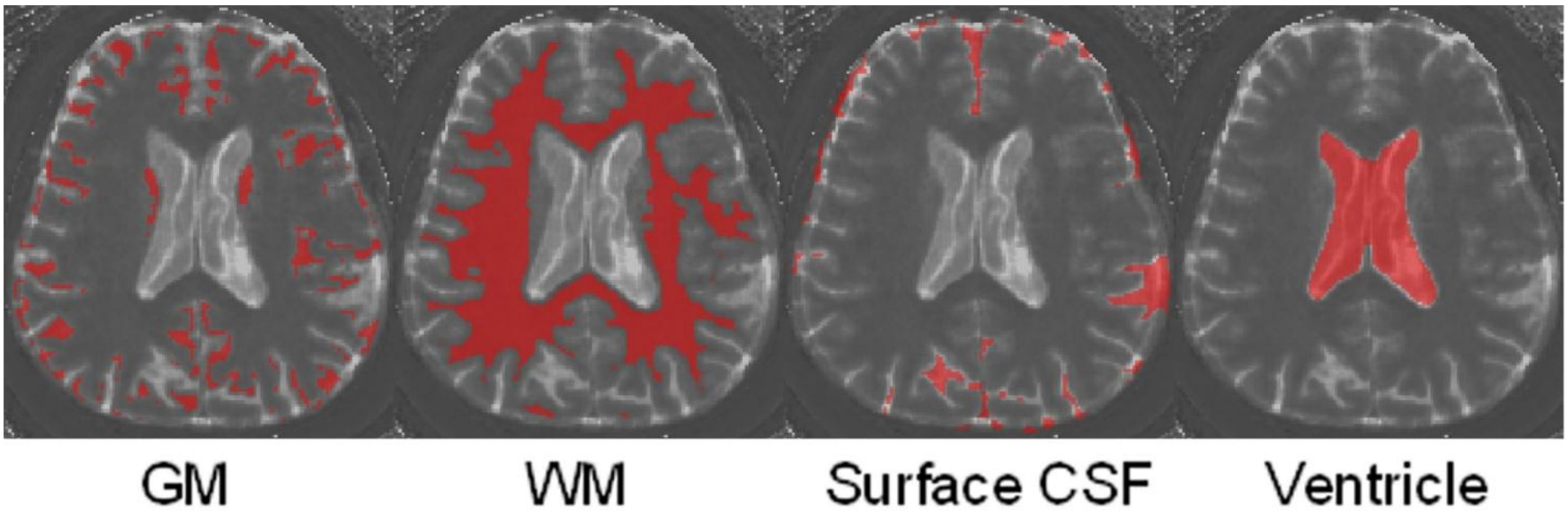


**Supplementary Fig. S2** VOIs eroded by 1 voxel from the original VOIs (shown in Supplementary Fig. S1) were additionally created. Representative images of 3D VOIs. *CSF* Cerebrospinal fluid, *GM* Gray matter, *VOI* Volume of interest, *WM* White matter.

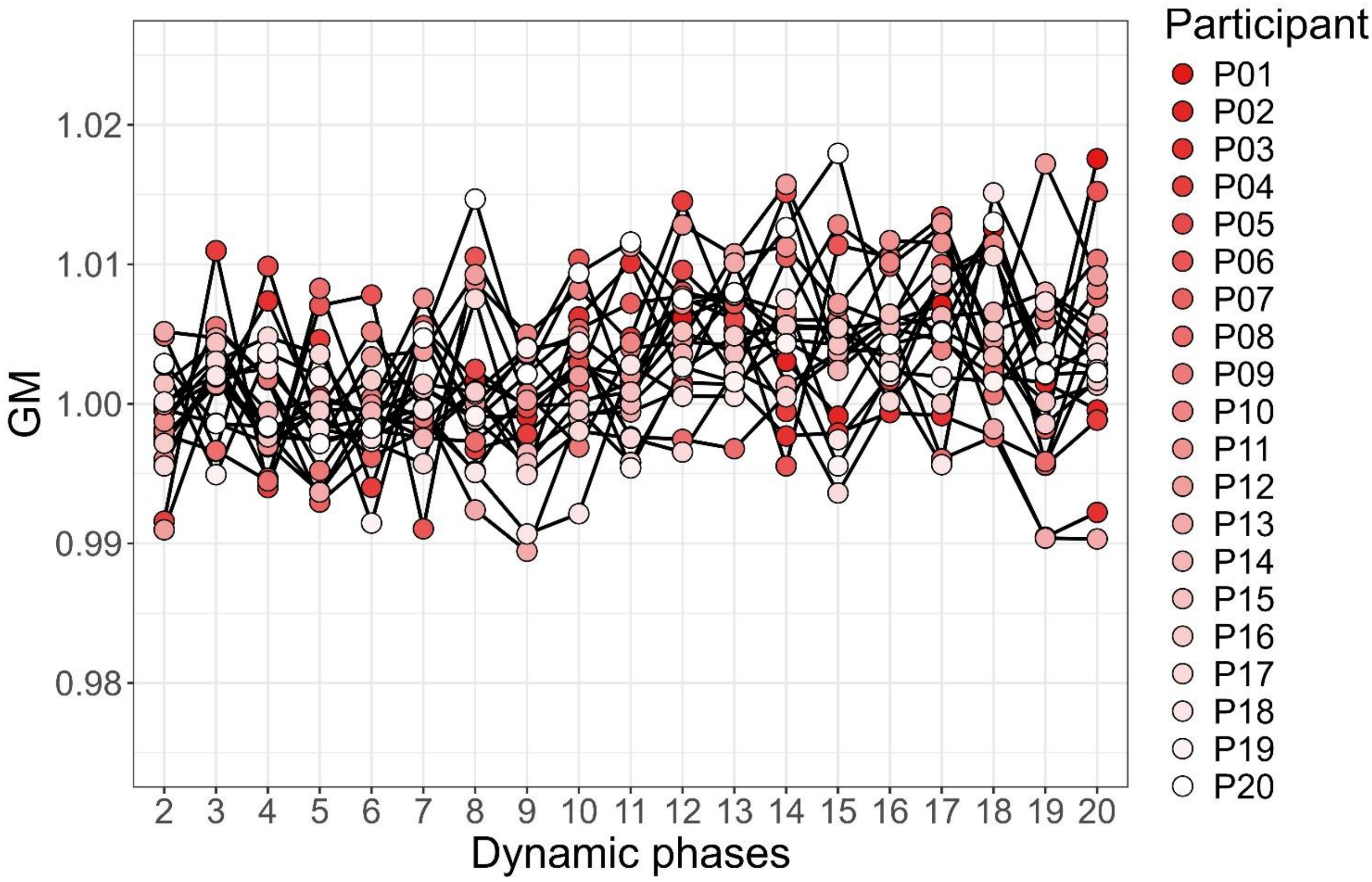


**Supplementary Fig. S3** Dynamic signal intensity changes on 3D VIBE for GM were presented with a thicker solid line and a narrower y-axis. A slight signal increase was also observed in GM. *GM* Gray matter, *VIBE* Volumetric Interpolated Breath-hold Examination.

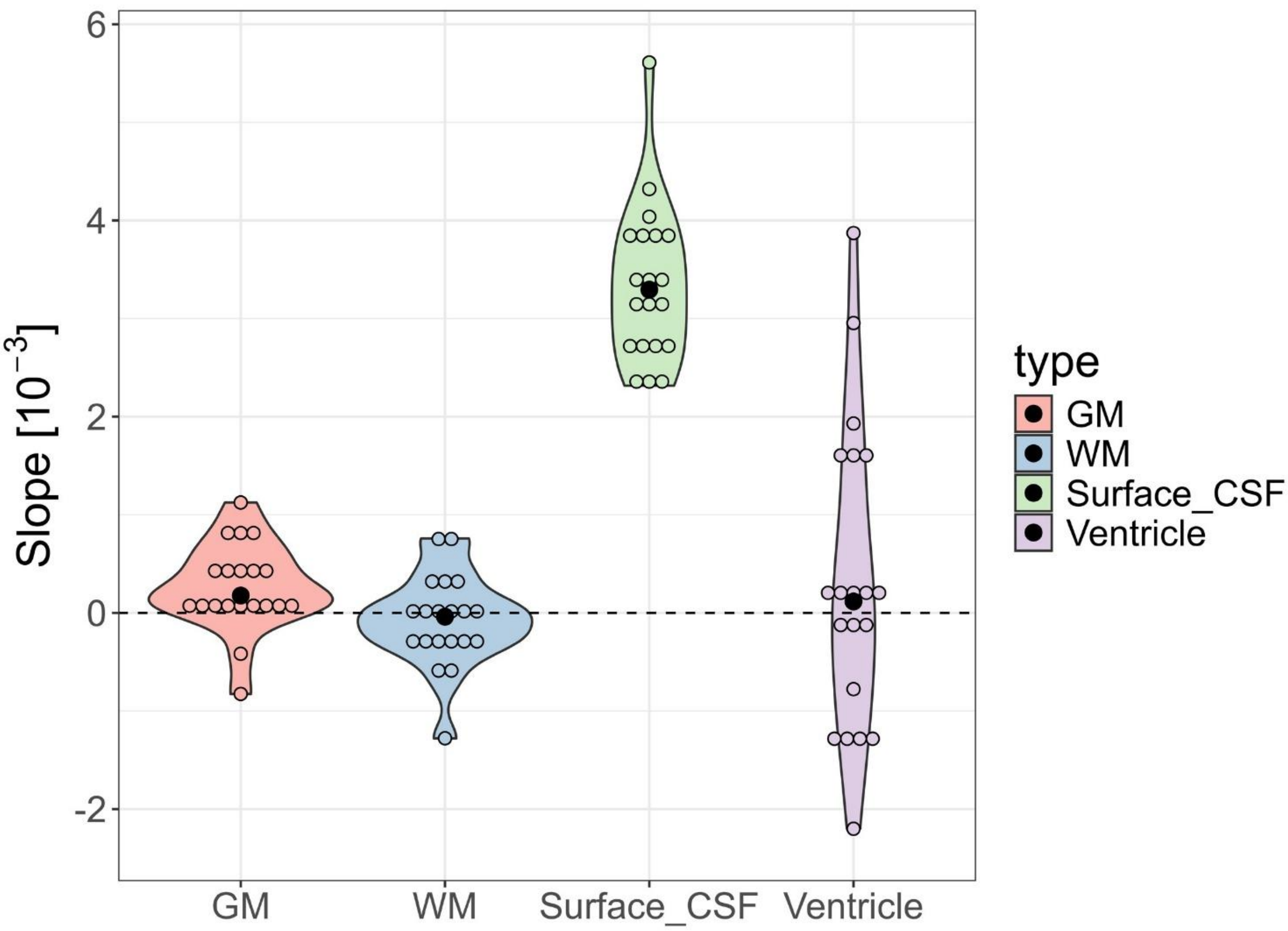


**Supplementary Fig. S4** Slopes of Post-$O_2$ VIBE (average of $VIBE_{9th}$ to $VIBE_{20th}$) were shown. Slope of surface CSF was significantly highest among four VOIs. *CSF* Cerebrospinal fluid, *VIBE* Volumetric Interpolated Breath-hold Examination, *VOI* Volume of interest.

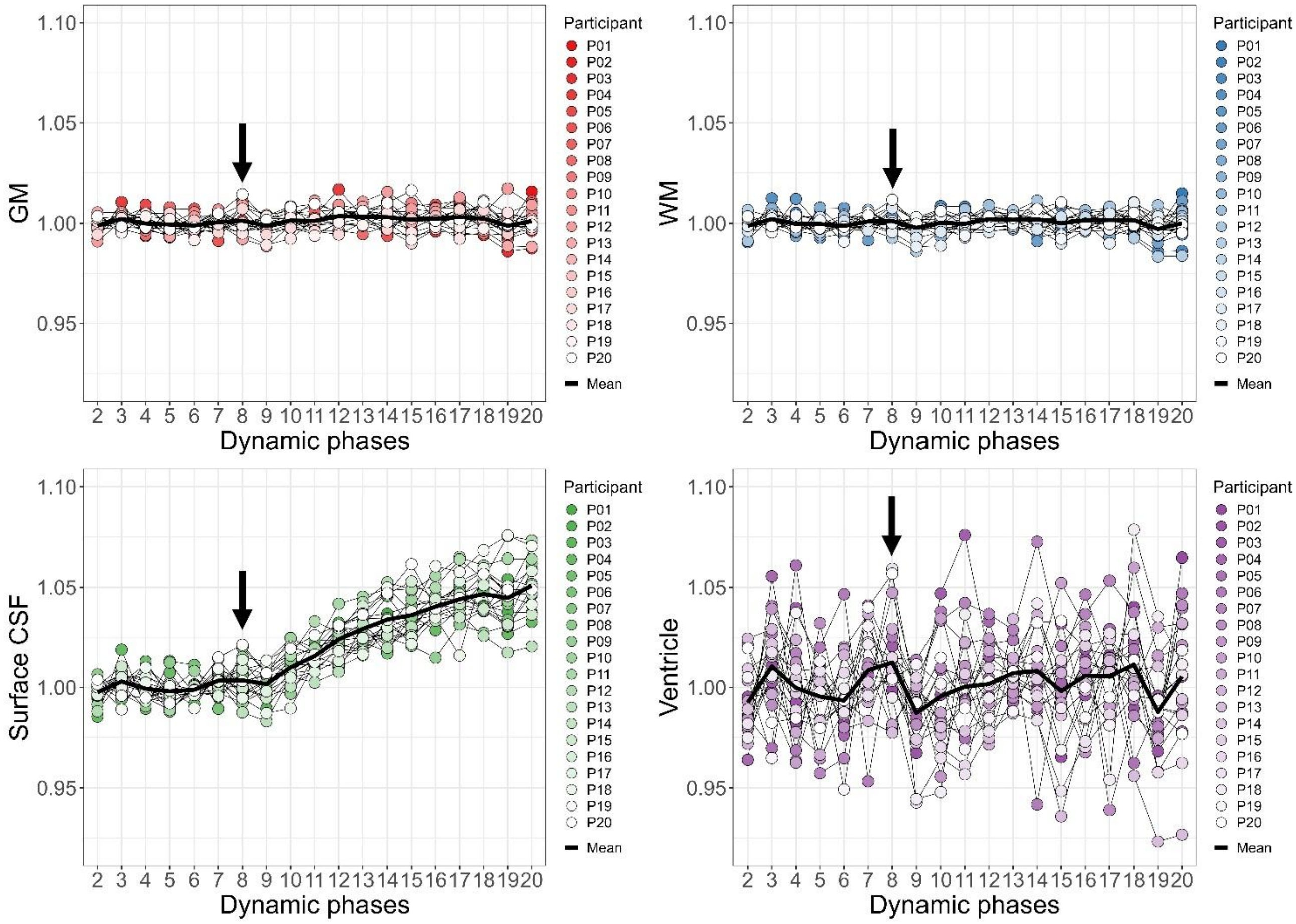


**Supplementary Fig. S5** Dynamic signal intensity changes on 3D VIBE using VOIs eroded by 1 voxel from the original VOIs. Temporal changes in the signal intensity ratio of $VIBE_{Nst}/VIBE_{2nd}$ are shown across VOIs. A rapid increase in signal was observed in the surface CSF immediately after $O_2$ inhalation. A slight signal increase was also observed in GM. Each circle represents the corresponding Participant 1-20. *CSF* Cerebrospinal fluid, *GM* Gray matter, *VIBE* Volumetric Interpolated Breath-hold Examination, *VOI* Volume of interest.

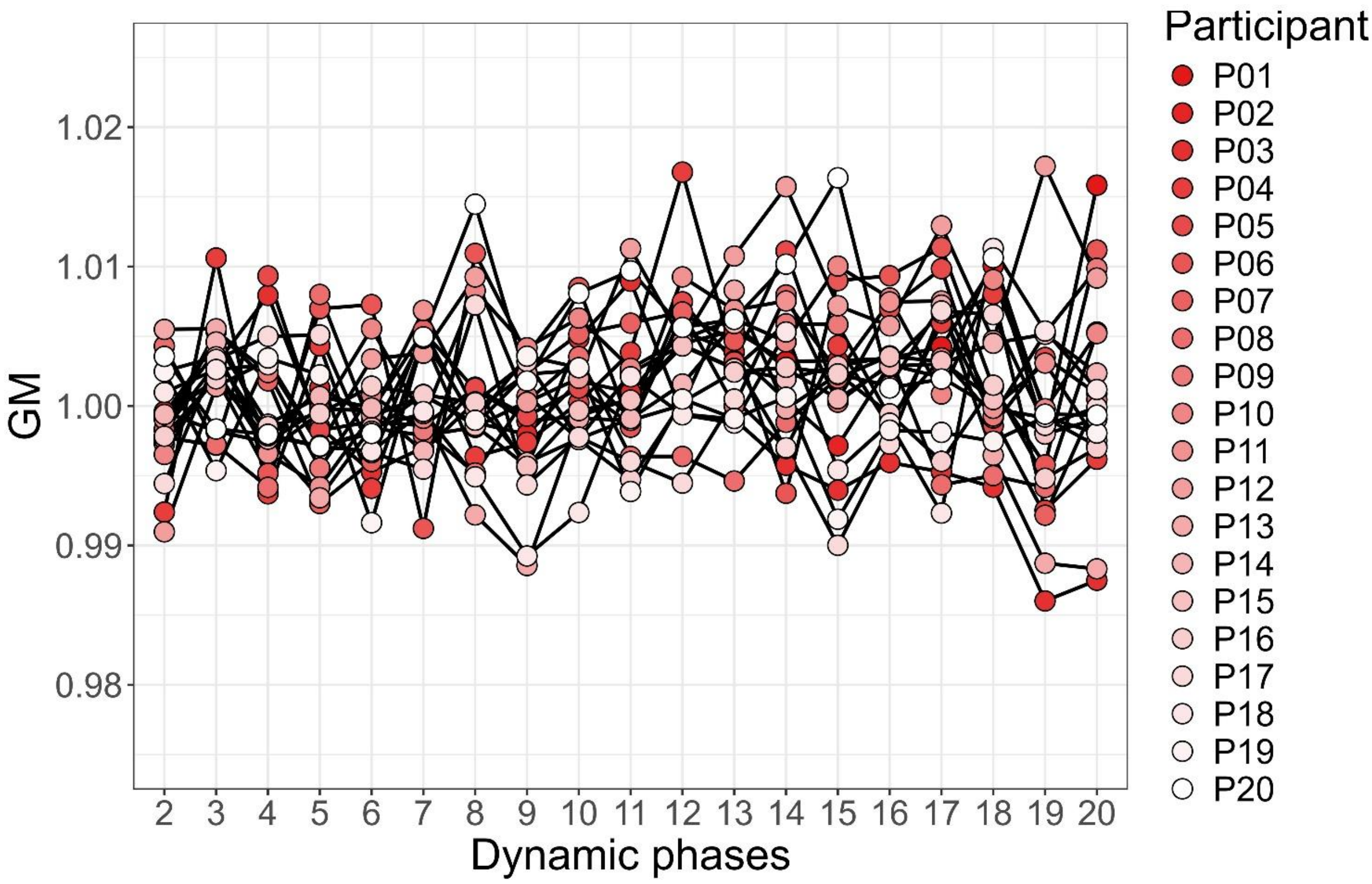


**Supplementary Fig. S6.** Dynamic signal intensity changes on 3D VIBE using VOIs eroded by 1 voxel from the original VOIs for GM were presented with a thicker solid line and a narrower *y*-axis. A slight signal increase was also observed in GM. *GM* Gray matter, *VIBE* Volumetric Interpolated Breath-hold Examination, *VOI* Volume of interest.

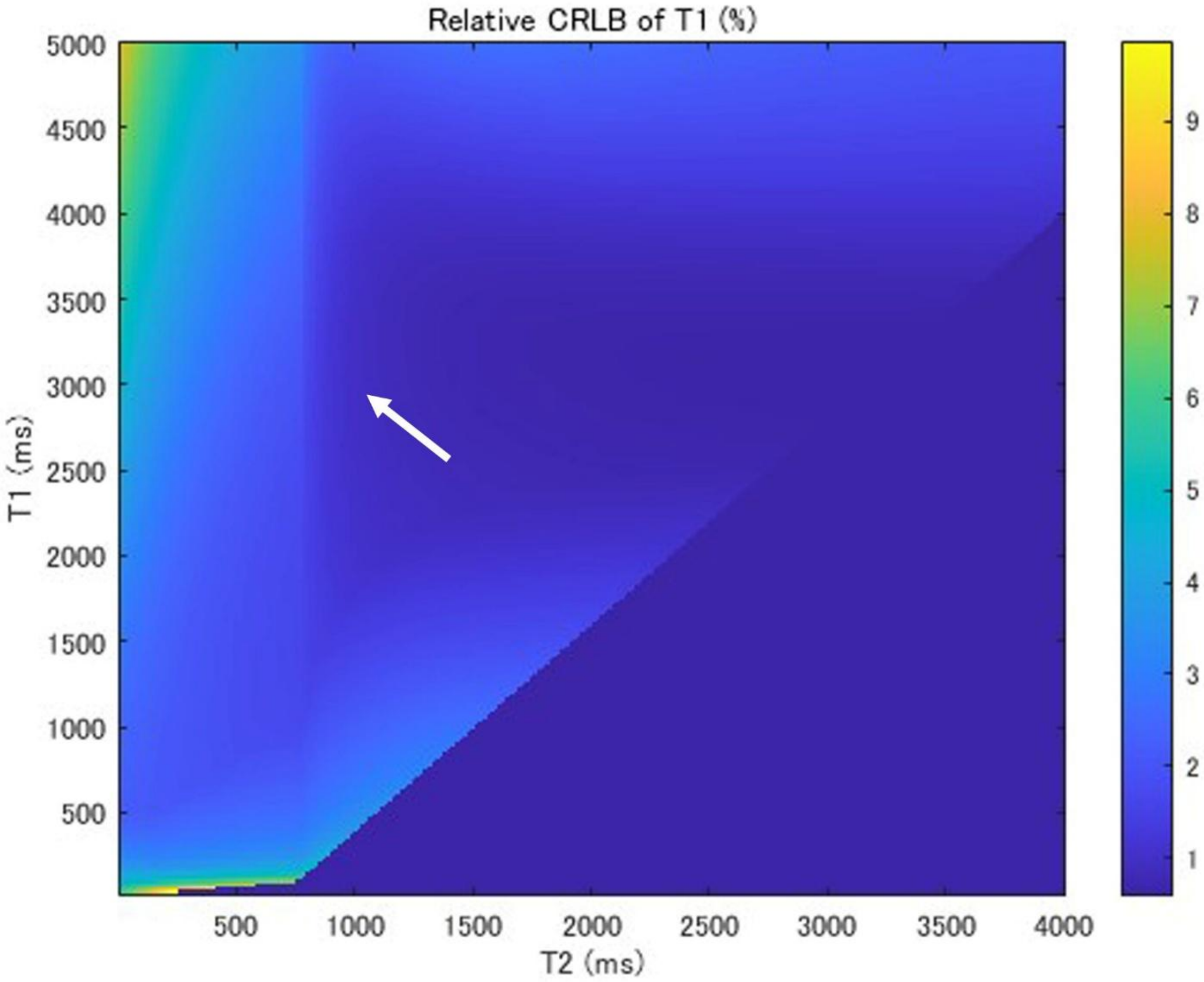


**Supplementary Fig. S7** Cramér–Rao Lower Bound (CRLB)-based sensitivity analysis using the current sequence was performed to evaluate precision over a range of T1/T2 values, including those typical of CSF. In general, lower CRLB values indicate more precise parameter estimation. To provide a quantitative anchor for the long-T1 regime characteristic of surface CSF, the theoretical precision floor at T1 = 3,000 ms and T2 = 1,000 ms using a conservative target SNR of 10 was shown. Under these conditions, the lower bound for T1 estimation error, CRLB_T1, was 21.26 ms, corresponding to a relative CRLB of 0.71%. The observed Post *minus* Pre T1 reductions in surface CSF were approximately 150–250 ms, which are about 7–12 times larger than this CRLB-based precision floor. These results indicate that the observed T1 reductions substantially exceed the expected uncertainty from estimation noise and are unlikely to be explained by numerical precision limits alone.
